# Thyrostimulin Regulates Osteoblastic Bone Formation During Early Skeletal Development

**DOI:** 10.1210/en.2014-1943

**Published:** 2015-05-27

**Authors:** J. H. Duncan Bassett, Anne van der Spek, John G. Logan, Apostolos Gogakos, Jayashree Bagchi-Chakraborty, Elaine Murphy, Clementine van Zeijl, Jenny Down, Peter I. Croucher, Alan Boyde, Anita Boelen, Graham R. Williams

**Affiliations:** Molecular Endocrinology Laboratory (J.H.D.B., J.G.L., A.G., J.B.C., E.M., G.R.W.), Department of Medicine, Imperial College London, London, W12 0NN United Kingdom; Department of Endocrinology (A.v.d.S., C.v.Z., A.Boe.), Academic Medical Centre, University of Amsterdam, 1100 DD Amsterdam, The Netherlands; Bone Biology Program (J.D., P.I.C.), Garvan Institute of Medical Research, Sydney, NSW 2010 Australia; and Centre for Oral Growth and Development (A.Boy.), Queen Mary, University of London, London, E1 4NS United Kingdom

## Abstract

The ancestral glycoprotein hormone thyrostimulin is a heterodimer of unique glycoprotein hormone subunit alpha (GPA)2 and glycoprotein hormone subunit beta (GPB)5 subunits with high affinity for the TSH receptor. Transgenic overexpression of GPB5 in mice results in cranial abnormalities, but the role of thyrostimulin in bone remains unknown. We hypothesized that thyrostimulin exerts paracrine actions in bone and determined: 1) *GPA2* and *GPB5* expression in osteoblasts and osteoclasts, 2) the skeletal consequences of thyrostimulin deficiency in GPB5 knockout (KO) mice, and 3) osteoblast and osteoclast responses to thyrostimulin treatment. *Gpa2* and *Gpb5* expression was identified in the newborn skeleton but declined rapidly thereafter. *GPA2* and *GPB5* mRNAs were also expressed in primary osteoblasts and osteoclasts at varying concentrations. Juvenile thyrostimulin-deficient mice had increased bone volume and mineralization as a result of increased osteoblastic bone formation. However, thyrostimulin failed to induce a canonical cAMP response or activate the noncanonical Akt, ERK, or mitogen-activated protein kinase (P38) signaling pathways in primary calvarial or bone marrow stromal cell-derived osteoblasts. Furthermore, thyrostimulin did not directly inhibit osteoblast proliferation, differentiation or mineralization in vitro. These studies identify thyrostimulin as a negative but indirect regulator of osteoblastic bone formation during skeletal development.

Glycoprotein hormones have a central role in regulation of the hypothalamic-pituitary-thyroid and hypothalamic-pituitary-gonadal axes. The glycoprotein hormones TSH, LH, FSH, and chorionic gonadotropin (CG) are heterodimeric proteins formed by the noncovalent association of a common α-subunit and a unique β-subunit that confers hormonal specificity. Thyrostimulin is a novel glycoprotein hormone recently identified as a result of homology to the cysteine-knot motif characteristic of the glycoprotein hormone and TGF-β superfamilies (1). In contrast to the known glycoprotein hormones thyrostimulin is formed from a heterodimer of unique α- and β-subunits, glycoprotein hormone subunit alpha (GPA)2 and glycoprotein hormone subunit beta (GPB)5, and is highly conserved across species.

Glycoprotein hormones act via type A leucine-rich repeat-containing G protein-coupled receptors (2, 3) and recombinant thyrostimulin has been shown to have high affinity for the TSH receptor (TSHR) (1). Indeed, human thyrostimulin is 4-fold more potent than human TSH at inducing a TSHR-mediated cAMP response (1, 4), whereas glycosylation of both GPA2 and GPB5 subunits are essential for secretion and activity of thyrostimulin (5, 6). Despite this, 100-fold higher concentrations of GPB5 alone can also induce a TSHR-mediated cAMP response (4).

Phylogenetic studies demonstrated that *GPA2* and *GPB5* encode ancestral glycoprotein hormone subunits that originated before the divergence of nematodes, arthropods and vertebrates more than 1 billion years ago (3, 7–9). *GPA2* and *GPB5* are, therefore, present in both vertebrates and invertebrates and thought to have given rise to the vertebrate common α-subunit (glycoprotein hormone α) and unique β-subunits (TSHβ, LHβ, and FSHβ) by gene duplication (10, 11).

Thyrostimulin expression has been investigated in several species (1, 4, 7, 12–14), and, in common with other glycoprotein hormones, GPA2 and GPB5 were initially found to be coexpressed in the pituitary (1, 4, 13). However, studies in *Drosophila melanogaster*, *Caenorhabditis elegans*, *Amphioxus*, and mouse indicated a broader developmental role for GPA2 and GPB5 (5, 15–17) and also suggested the subunits might act independently as monomers or homodimers (5). Furthermore, GPB5 lacks the conserved “seatbelt” motif considered necessary for glycoprotein hormone heterodimer stability in the circulation. Taken together, these findings indicate that GPA2/GPB5 heterodimers may predominantly exist at local sites of coexpression and thus suggest a paracrine rather than endocrine role for thyrostimulin (7, 18). Accordingly, coexpression of GPA2 and GPB5 has been documented in various tissues and paracrine roles for thyrostimulin have been proposed in the pituitary (1, 4, 17), ovary (19), and skin (20).

Targeted manipulation of *GPA2* and *GPB5* expression in mice has, thus far, yielded only limited insight into the physiological role of thyrostimulin. Initial analysis of thyrostimulin-deficient animals (GPB5 knockout [KO]) did not identify an overt phenotype (4, 21). Nevertheless, juvenile mutants were subsequently found to have isolated mild hypothyroxinaemia and abnormal responses to thyroid hormone manipulation (17). By contrast, global overexpression of GPB5, from the *Rosa26* locus, resulted in a 2-fold increase in systemic thyroid hormone levels, resistance to diet-induced obesity, increased metabolic rate and abnormal skull development (21). Similarly, transgenic overexpression of GPB5 under control of the metallothionein-1 promoter resulted in a 3-fold elevation of serum T_4_ and thyroid follicular and adrenocortical cell hyperplasia (4). This phenotype was replicated in wild-type (WT) mice, by a once daily ip injection of recombinant GPA2/GPB5 (4). By contrast, transgenic over expression of GPA2 resulted in no phenotype (4). Overall, these studies have been interpreted to indicate that thyrostimulin is not essential for mammalian development and reproduction, and that most of the phenotypic consequences of thyrostimulin excess are secondary to activation of the thyroid axis (4, 21).

In summary, the roles of the ancestral glycoprotein hormone “thyrostimulin” and its unique subunits GPA2 and GPB5 are unknown, and, despite demonstration of TSHR expression in bone cells (22–24), the skeletal-consequences of GPB5 overexpression remain unexplained (21). We hypothesized that thyrostimulin exerts paracrine actions in the skeleton. To investigate this hypothesis we determined *GPA2* and *GPB5* expression in primary osteoblasts and osteoclasts, analyzed the skeletal phenotype of thyrostimulin-deficient mice, and determined osteoblast and osteoclast responses to thyrostimulin treatment.

## Materials and Methods

### Cell culture

#### Mouse osteoblast culture

Primary osteoblasts were prepared from calvariae of postnatal day (P)3 C57BL/6 mice (25). Osteoblasts were cultured in the absence or presence of T_3_ (100nM). Cells were harvested for RNA extraction on days 6, 9, 12, and 28. Mouse osteoblastic MC3T3-E1 cells (ATCC CRL-2593) were cultured as described (26).

Osteoblast proliferation, differentiation and mineralization experiments were performed using primary mouse calvarial osteoblasts seeded at 10^6^ cells/well in α Modified Eagle Medium (MEM) supplemented with 10% fetal calf serum (FCS), penicillin and streptomycin (PS), β-glycerol phosphate (2.5mM), and L-ascorbic acid (50 μg/mL). Cells were treated for 9 or 28 days with medium alone (control) or supplemented with conditioned medium from COS-7 cells (10% vol/vol) transfected with empty vector (vector), conditioned medium from COS-7 cells (10% vol/vol) transfected with GPA2/GPB5 expression vector (GPA2/GPB5), or with PTH (100μM).

#### Mouse bone marrow stromal cell (BMSC)/osteoblast culture

Primary BMSC/osteoblast cultures were prepared as described (27). Bone marrow was flushed from long bones of 8-week-old C57BL/6 mice and seeded at 500 000 cells/cm^2^ in αMEM supplemented with 10% FCS containing PS. At 70% confluence, osteoblast differentiation was induced by addition of β-glycerol phosphate (5mM) and ascorbate phosphate (50 μg/mL). Osteoblast proliferation, differentiation and mineralization experiments were performed in cells treated for 9 or 28 days as above.

#### Human osteoblast culture

Normal human osteoblasts (Clonetics) were seeded at 1 × 10^4^ cells/cm^2^ and cultured until confluent in αMEM containing 10% heat-inactivated FCS (HI-FCS) containing PS. Cells were reseeded at 1 × 10^4^ cells/cm^2^ and differentiated in αMEM containing HI-FCS (10%) and PS supplemented with hydrocortisone-21-hemisuccinate (200nM), 55-μg/mL ascorbic acid and 7.5mM β-glycerophosphate (Clonetics) in the absence or presence of T_3_ (100nM) at 37°C in 5% CO_2_. Cells were harvested for RNA on days 7, 14, 21, and 28.

#### Mouse osteoclast culture

Bone marrow osteoclast progenitors were prepared from P35 C57BL/6 mice (24). Bone marrow was flushed from long bones, resuspended in αMEM containing FCS (10% vol/vol) and M-CSF (25 ng/mL) (R&D Systems), plated at 1.5 × 10^6^ cells/mL, and cultured overnight. Nonadherent cells were replated at 1 × 10^6^ cells/mL in αMEM containing FCS (10%), macrophage colony stimulating factor (M-CSF) (25 ng/mL) and receptor activator of nuclear factor kappa-B ligand (10 ng/mL) (R&D Systems) and cultured ±T_3_ (100nM). Cells were harvested for RNA on days 6, 9, and 12. Some cultures were stained for tartrate-resistant acid phosphatase (TRAP) activity (Sigma-Aldrich) to confirm the presence of osteoclasts.

#### Human osteoclast culture

A total of 40-mL peripheral blood collected with EDTA was diluted 1:1 with PBS, and mononuclear cells were isolated using a LeucoSep centrifuge tube (Greiner bio-one) containing 15 mL of histopaque (Sigma-Aldrich) according to manufacturer's instructions. Cells were resuspended in αMEM containing HI-FCS (10%), PS, M-CSF (25 ng/mL), and receptor activator of nuclear factor kappa-B ligand (50 ng/mL) (R&D Systems), plated at 3 × 10^6^ cells/mL, and cultured overnight. Nonadherent cells were removed and cultured at 1 × 10^6^ cells/mL ±T_3_ (100nM) at 37°C in 5% CO_2_. Cells were harvested for RNA on day 35. Osteoclast formation was confirmed by TRAP staining.

#### Chinese hamster ovary (CHO) cell culture

A CHO cell line stably transfected with human TSH receptor (JP26) and a control cell line with vector alone (JP02) were provided by Sabine Costagliola (28). Cells were maintained in Coon's modification of Ham's medium (F-12 medium) with L-glutamine (Invitrogen), supplemented with 10% FCS, PS, and Geneticin (G418 400 μg/mL) (Invitrogen) at 37°C in 5% CO_2_.

### RNA isolation and RT-PCR

Total RNA was extracted using TRIzol (Invitrogen) according to manufacturer's instructions. Fresh bone samples were pulverized at −80°C using a steel pestle and mortar (Biospec), the resulting powder was homogenized in TRIzol and RNA extracted. RT-PCR was performed using cDNA synthesized with polyA primers and superscript II reverse transcriptase (Invitrogen). A total of 1-μg total RNA was denatured at 70°C for 10 minutes, polyA primers and 1 U of superscriptase II were added and incubated for a further 30 minutes at 42°C. PCR was performed using Platinum Taq DNA polymerase (Invitrogen).

Expression of osteoblast marker genes *Runx2*, osterix (*Osx*), and osteocalcin (*Oc*) was determined in calvarial osteoblasts and BMSC/osteoblasts. Cells were lysed in TRIzol and RNA isolated using an RNeasy kit (QIAGEN). RNA quantity and quality was determined using a Nanodrop 1000 spectrophotometer (Thermo Scientific). A total of 750-ng RNA was converted to cDNA using a Quantitect reverse transcription kit (QIAGEN) and used for real-time PCR using a KAPA SYBR Fast qPCR kit (KAPA Biosystems). Reactions were run on a 7900HT real-time PCR system (Applied Biosystems): 50°C for 2 minutes, 95°C for 10 minutes followed by 40 cycles with 95°C for 30 seconds, 60°C for 30 seconds, and 72°C for 30 seconds. Samples from 3 independent experiments were run in triplicate; results were calculated by comparison with a standard curve and presented as arbitrary units relative to 18S rRNA. Primers are detailed in Supplemental Table 1.

### GPB5KO and WT mice

GPB5KO mice (129SvEv background) were generated by Lexicon Genetics, Inc. Heterozygous GPB5^+/−^ mice were crossed for 3 generations before maintenance of separate WT and GPB5KO breeding lines (17). Germline transmission of the knockout allele and genotyping was confirmed by PCR using specific primers (Common-forward, GCCTGAGACTGCTTTGAGGG; WT-reverse, CCACTGCACCAAGGACAAGG; and GPB5KO-reverse, CTGCCTGACTTCCTCACAGC) (Sigma REDExtract-N-Amp Tissue PCR kit; Sigma-Aldrich). Animals were maintained at 22°C with a 12-hour light, 12-hour dark cycle with ad libitum food and water. Male and female GPB5KO and WT mice were analyzed at P42 (6 wk) and P112 (16 wk). Tail lengths were determined during growth and at killing. Mice received 2 injections of calcein (15 mg/kg in saline with 2% NaHCO_3_) 7 and 4 days before killing. The local animal welfare committee approved the study, and animals were treated according to ETS123 (Council of Europe) guidelines.

### Thyroid function

#### Serum thyroid hormone levels

Serum T_4_ and T_3_ were measured using in-house RIAs, and TSH was determined using a double-antibody precipitation RIA (17).

#### TRH test

At P42, mice were injected ip with 100-ng TRH (Ferring) in 0.5-mL sterile 0.9% NaCl, or with vehicle (29). Serum was collected before and 20 minutes after TRH administration, and TSH levels were determined (17).

### Histological analysis of the growth plate

Limbs were fixed in 10% neutral buffered formalin for 24 hours and decalcified in 10% EDTA for 5 days. Paraffin-embedded sections were stained with van Gieson and Alcian blue, and growth plate parameters were determined as described (30). Images of the proximal tibia were acquired using a Leica DM LB2 microscope (Leica AG) and DFC320 digital camera. Measurements at 4 locations across the width of growth plates were obtained using ImageJ (http://rsb.info.nih.gov/ij/) to calculate mean heights of the reserve, proliferative and hypertrophic zones, and total growth plate. Results from 2 midline levels of sectioning were compared to ensure data consistency.

### Skull microscopy

Skulls from P42 mice were imaged using a Leica MZ75 binocular microscope, KL1500 light source, and DFC320 digital camera. Skull dimensions were determined using ImageJ.

### Digital x-ray microradiography

Skin and soft tissue was removed from lower limbs and digital x-ray images recorded at 10-μm resolution using a Faxitron MX20 variable kV point projection x-ray source and digital image system operating at 26 kV and ×5 magnification (Qados, Cross Technologies plc). Relative mineral content was determined as described (31). Superior and lateral skulls images were obtained at 30 kV and dimensions determined using ImageJ.

### Micro-computerized tomography (CT) analysis

Long bones were analyzed by micro-CT (Skyscan 1172a; Bruker) at 50 kV and 200 mA using a 0.5-mm aluminum filter and a detection pixel size of 4.3 mm^2^ as described (30, 32). Trabecular bone parameters (gray level thresholds 85 and 255) were determined in a 1-mm^3^ region of interest (ROI) 0.2 mm from the growth plate (33). Cortical bone parameters (gray level thresholds 85 and 255) were determined in a 1-mm ROI in the middiaphysis (33). To generate high-resolution three-dimensional images, 16-bit Tiff images were imported into 32-bit Drishti (Australian National University Supercomputer Facility, http://anusf.anu.edu.au/Vizlab/drishti/), and ROIs were rendered using 64-bit Drishti.

### Back scattered electron (BSE)-scanning electron microscopy (SEM)

Femurs and tibias were fixed in 70% ethanol, opened longitudinally and macerated as described (34). Samples were coated with carbon and imaged using backscattered electrons at 20-kV beam potential in a Zeiss DSM962 digital scanning EM equipped with an annular solid state BSE detector (KE Electronics).

### Quantitative BSE-SEM (qBSE-SEM)

The distribution of mineralization densities within calcified tissues was examined by BSE-SEM quantified by digital image analysis at the cubic micron volume resolution scale. Humeri were fixed in 10% neutral buffered formalin for 24 hours and transferred to 70% ethanol before embedding in poly-methyl-methacrylate. Longitudinal block faces were cut through specimens, which were then polished, coated with carbon, and analyzed using backscattered electrons in a Zeiss DSM962 operated at 20 kV and 0.5 nA and a 17-mm working distance (11 mm, sample to detector distance). The mineralization densities of calcified tissues were determined relative to halogenated dimethacrylate standards: C_22_H_25_O_10_Br (mean BSE coefficient = 0.1159) to C_22_H_25_O_10_I (mean BSE coefficient = 0.1519). Increasing gradations of micromineralization density were represented in 8 equal intervals by a pseudocolor scheme (34).

### Confocal microscopy analysis of bone formation

Two-dimensional parameters of bone formation were determined according to the American Society for Bone and Mineral Research system of nomenclature (35). Midline longitudinal block faces were cut through poly-methyl-methacrylate-embedded long bones. Specimens were polished to an optically flat finish and examined using confocal autofluorescence scanning light microscopy (CSLM) of calcein labels to determine the fraction of bone surface undergoing active bone formation (n = 3–5 mice per genotype, per gender). A Leica SP1 confocal microscope using 488-nm excitation and ×40/1.25 objectives generated combined reflection (blue), calcein fluorescence (green), and autofluorescence (red) CSLM images (30). Tissue layers immediately deep to the block surface were visualized to ensure only fluorescently labeled bone-forming surfaces that lie in orthogonal planes were analyzed. The mineral apposition rate (MAR) was calculated by determining calcein separation at 10–20 locations per specimen. Montages were generated, and the bone and mineralizing surfaces measured using ImageJ (30). Montages of 20 (magnification ×40) overlapping CSLM fields were constructed for each bone. Trabecular and endosteal bone surfaces were determined using ImageJ. Mineralizing surface and MAR were determined by quantifying calcein-labeled surfaces and the mean separation between calcein double labels. Bone formation rate (BFR) was calculated from the product of mineralizing surfaces and MAR. Measurements commenced 200 μm (trabecular bone) and 2 mm (cortical bone) below the growth plate (30).

### Osteoclast histomorphometry

Osteoclast numbers were determined according to the American Society for Bone and Mineral Research system (35) in paraffin sections stained for TRAP activity using a Sigma TRAP kit (386A-1KT) according to manufacturer's instructions. For each sample sections from 2 separate levels (n = 2 slides) were photographed at ×100 magnification using a Leica DMLB2 microscope and DFC320 digital camera, and a 750 × 750-μm ROI commencing 250 μm below the growth plate was analyzed. Osteoclast numbers per bone perimeter (Oc.N/B.Pm), osteoclast perimeter (Oc.Pm), and osteoclast surface per bone perimeter (Oc.S/B.Pm) were determined in trabecular bone (n = 3–5 mice per genotype, per gender) and normalized to total bone perimeter (B.Pm) (30). The fraction of the bone surfaces that displayed osteoclastic bone resorption was quantified in high-resolution BSE-SEM images (30).

### Destructive 3-point bend testing

Bones were stored and tested in 70% ethanol. Destructive 3-point bend tests were performed using an Instron 5543 load frame and 100N load cell (Instron). Humeri were positioned horizontally on custom supports and load was applied perpendicular to the middiaphysis with a constant rate of displacement of 0.03 mm/s until fracture. Yield load, maximum load, fracture load, and stiffness were determined from load displacement curves (30, 32).

### Expression of GPA2/GPA5

#### Gpa2 and Gpb5 expression vectors

Full-length Gpa2 and Gpb5 cDNAs were amplified from total mouse brain RNA by RT-PCR using primers mGPA2F, GACTGTCCTTTGCAGATGCCC and murine GPA2R, AGCCCCGAG TTTGAGATACCC for *Gpa2* and primers mGPB5F, AGCCTGGGGTACAAGTGTCAGC and mGPB5R, TGGAGCCAGTGGATGTGTGAG for *Gpb5*. The *Gpa2* and *Gpb5* cDNAs were subcloned into pGEM-T Easy (Promega) and sequenced (36). GPA2 and GPB5 subunits were either coexpressed or expressed individually using the bicistronic vector pBudCE4.1 (Invitrogen): 1) *Gpa2* was subcloned into the pBudCE4.1 using the *Not*I and *Xho*I sites resulting in expression of a His-V5-tagged GPA2 protein (GPA2-His-V5), 2) *Gpb5* was subcloned into pBudCE4.1 using the *Hin*dIII and *Bam*HI sites resulting in expression of a hemagglutinin (HA)-tagged GPB5 protein (GPB5-HA), and 3) both *Gpa2* and *Gpb5* were subcloned into pBudCE4.1 (GPA2-His-V5+GPB5-HA).

#### Transient transfection of Cos-7 cells

5 × 10^6^ Cos-7 cells were cultured overnight in DMEM (Invitrogen), 10% HI-FCS, containing PS. Cells were transfected with 1) GPA2-His-V2, 2) GPB5-HA, 3) GPA2-His-V2+GPB5-HA, or 4) pBudCE4.1 vector alone using the Lipofectamine 2000 reagent (Invitrogen) according to manufacturer's instructions and cultured for 72 hours. Medium was concentrated 20-fold using a centrifugal filter and cell lysates prepared in radioimmunoprecipitation assay buffer (Sigma-Aldrich).

#### Western blot analysis

Medium and cell lysates were subjected to SDS-PAGE. Membranes were blocked with fat-free milk (5%) with Tween (1%) for 1 hour and incubated overnight at 4°C in the presence of either a monoclonal mouse anti-V5 (Sigma-Aldrich) or anti-HA (Invitrogen) antibody. Blots were visualized using a goat antimouse horseradish peroxidase-conjugated antibody and SuperSignal West Femto enhanced chemiluminescent substrate (Thermo Scientific) according to manufacturer's instructions. Conditioned media were deglycosylated using N-glycosidase F (New England Biolabs) or covalently cross-linked with disuccinimidyl suberate (Sigma-Aldrich), according to manufacturer's instructions, and the products analyzed by Western blotting.

#### cAMP immunometric assay

cAMP assays were performed in confluent JP02 and JP26 CHO cells. Medium was replaced with Krebs-Ringer HEPES (KRH) buffer, and cells were incubated for 30 minutes at 37°C. KRH buffer was replaced with 1) KRH containing rolipram (25μM) (Sigma), which prevents degradation of cAMP; 2) KRH containing rolipram (25μM) and forskolin (10μM) (Sigma); 3) KRH containing rolipram (25μM) and human TSH (hTSH) (0.001, 0.01. and 0.1 U/L); or 4) conditioned media from Cos-7 cells transfected with the various constructs (500 μL) containing rolipram (25μM). Cells were incubated at 37°C for 1 hour, medium was removed, and cells were lysed with 0.1M HCl for 20 minutes. cAMP was quantified in 100 μL of cell lysate using cAMP EIA kit (Stressgen Biotechnologies Corp) following manufacturer's instructions.

#### Noncanonical TSHR signaling pathway analyses

Activation of noncanonical signaling pathways was determined in cells cultured for 9 or 28 days. Cells were incubated in serum-free αMEM overnight before treatment for 5, 15 or 30 minutes with 1) αMEM medium control, 2) conditioned medium from COS-7 cells transfected with empty vector (20% vol/vol), or 3) conditioned medium from COS-7 cells transfected with GPA2/GPB5 (20% vol/vol). Cells were lysed and the concentrations of phospho-AKT, phospho-ERK, and phospho-mitogen-activated protein kinase (P38) (P38 MAPK) determined according to the manufacturer's instructions (Surveyor IC phospho-kinase immunoassay kits [SUV887B, SUV1018B, SUV869B]; R&D Systems).

#### Osteoblast proliferation, differentiation, and mineralization assays

The number of viable osteoblasts was determined using the alamarBlue assay (Life Technologies). Osteoblast differentiation was quantified by alkaline phosphatase (ALP) activity normalized to cell number: cells were homogenized in 1M diethanolamine, 1mM MgCl_2_, 0.05% Triton X-100, the lysate was mixed with an equal volume of paranitrophenol-phosphate (20mM) and absorbance measured at 414 nm. Mineralization was determined by alizarin red staining: cells were fixed in 70% ethanol, stained for 20 minutes in 40mM alizarin red (pH 4.2), and destained for 15 minutes with acetylpyridinium chloride (10% wt/vol in 10mM Na_3_PO_4_). Absorbance of extracted alizarin red was measured at 562 nm and normalized to cell number.

### Statistics

Normally distributed data were analyzed by Student's *t* test and nonparametric data by the Mann-Whitney *U* test. *P* < .05 was considered significant. Frequency distributions of bone mineral densities obtained by Faxitron and qBSE-SEM were compared using the Kolmogorov-Smirnov test (30, 37).

## Results

### Expression of TSH, thyrostimulin, and TSHR in bone

**Figure 1.**
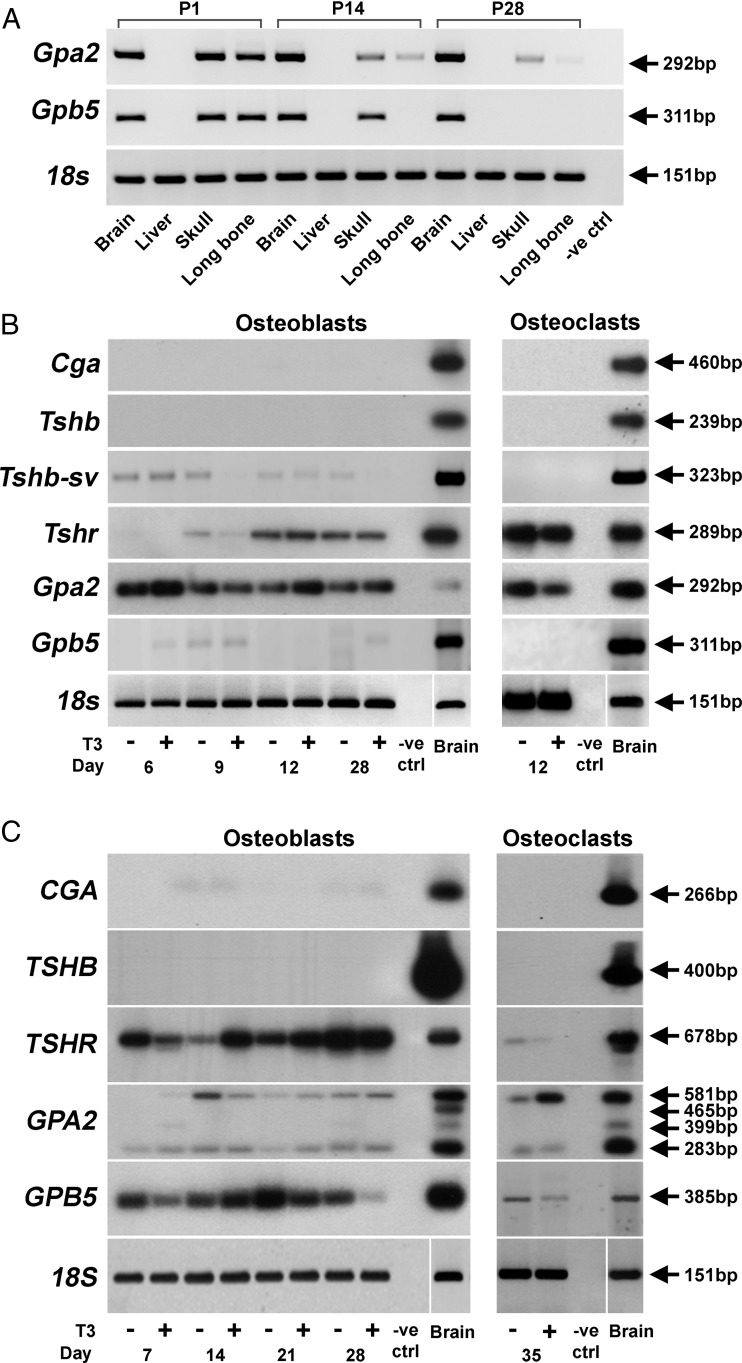
Mouse and human GPA2, GPB5, CGA, TSHB, and TSHR mRNA expression. A, *Gpa2* and *Gpb5* mRNA, and 18s rRNA, expression in skeletal and nonskeletal mouse tissues during development at P1, P14, and P28. −ve ctrl, no reverse transcriptase control. B, *Cga*, *Tshb*, *Tshr*, *Gpa2*, and *Gpb5* mRNA expression in mouse primary osteoblasts and osteoclasts cultured in the presence or absence of 100nM T_3_. C, *CGA*, *TSHB*, *TSHR*, *GPA2*, and *GPB5* mRNA expression in human primary osteoblasts and osteoclasts in the presence or absence of 100nM T_3_. Noncontiguous lanes are separated by a white line. *Cga*, glycoprotein hormones, α; *Tshb*, TSHβ; *Tshb-sv*, TSHβ splice variant; *Tshr*, TSHR; *Gpa2*, glycoprotein hormone α2; *Gpb5*, glycoprotein hormone β5.

Expression of both *Gpa2* and *Gpb5* mRNAs was readily identified in skulls and long bones from newborn WT mice. Expression of *Gpa2* was reduced by P14 and barely detectable at P28, whereas expression of *Gpb5* was only present in skulls at P14 but was absent from both skulls and long bones at P28 (Figure 1A). Neither *CGA* nor *TSHB* mRNAs (encoding the α- and β-subunits of TSH, the established hormone that binds and activates TSHR) were expressed in mouse or human primary osteoblast or osteoclast cultures (Figure 1, B and C). *TSHR* mRNA was expressed in mouse and human osteoblasts and mouse osteoclasts but was at the limit of detection in human osteoclasts. *GPA2* was expressed in both mouse and human osteoblasts and osteoclasts. Four distinct human *GPA2* RT-PCR products were amplified from brain mRNA but only 2 were expressed in osteoblasts and osteoclasts. Sequencing revealed the size differences resulted from retention of intron 2 (182 bp), intron 3 (116 bp), or both introns 2 and 3 (Supplemental Figure 1A). Inclusion of intron 2 results in an open reading frame terminated by a stop codon after 93 bp, whereas inclusion of intron 3 results in an open reading frame terminated by a stop codon after 69 bp. The *GPA2* mRNAs expressed in human osteoblasts and osteoclasts consisted of the full-length correctly spliced *GPA2* mRNA (283 bp) and an unspliced transcript (581 bp), in which introns 2 and 3 are retained (Supplemental Figure 1A). By contrast, *Gpb5* was expressed only at low levels early in differentiation in mouse osteoblasts but was not detected in mouse osteoclasts (Figure 1B), whereas *GPB5* mRNA expression was clearly detectable in human osteoblasts and osteoclasts (Figure 1C).

**Figure 2.**
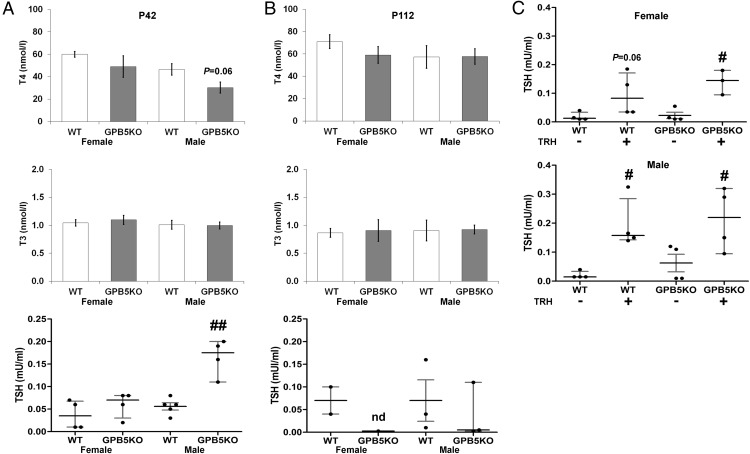
Serum thyroid hormone and TSH levels. A and B, Serum T_4_, T_3_, and TSH levels in female and male juvenile (P42) and adult (P112) WT and GPB5KO mice (mean ± SEM for T_4_ and T_3_; individual values, median, and interquartile range for TSH). Mann-Whitney *U* test; #, *P* < .05: ##, *P* < .01 GPB5KO vs WT (n = 4–9 per genotype per age). nd, not determined because insufficient sample. C, Serum TSH response to TRH (100 ng ip) in female and male juvenile (P42) WT and GPB5KO mice. Mann-Whitney *U* test; #, *P* < .05 response to TRH; GPB5KO+TRH vs WT+TRH not significant (n = 6–8 per genotype).

An alternative spice variant of *Tshb* (*Tshb-sv*) that includes a 27 nucleotide portion of intron 4 adjoining the coding region of exon 5 has been previously identified in mouse (Figure 2B) but not human bone marrow and has also been shown to stimulate a cAMP response in TSHR-expressing cells in vitro (38–41). The *Tshb-sv* isoform was present at low levels in primary mouse osteoblasts but not in osteoclasts (Figure 1B). Notably, this 27bp sequence has only limited conservation between species (Supplemental Figure 1, C and D).

T_3_ treatment did not affect expression of *CGA*, *TSHB*, *TSHR*, *GPA2*, or *GPB5* in either human or mouse primary cultures, suggesting that effects of T_3_ on bone are not mediated via modulation of TSHR signaling.

### Analysis of thyrostimulin-deficient mice

Having demonstrated that both subunits of thyrostimulin (*GPA2* and *GPB5*) were expressed in whole bone but variably expressed in skeletal cells, we next investigated the physiological role of thyrostimulin during skeletal development and in adult bone by determining the consequences of thyrostimulin deficiency in mice lacking *Gpb5* (GPB5KO).

### Systemic thyroid status in thyrostimulin-deficient mice

At P42, there was no difference in thyroid status between female WT and GPB5KO mice, whereas male GPB5KO mice had a 3-fold increase (*P* < .01) in circulating TSH, a 35% lower T_4_ (*P* = .06), but a normal T_3_ concentration compared with WT. Nevertheless, administration of TRH resulted in similar TSH responses in both WT and mutant animals. Adult female and male GPB5KO mice were euthyroid (Figure 2).

### Increased bone mineral content (BMC), volume, and mineralization in juvenile thyrostimulin-deficient mice

#### Normal ossification and linear growth

Growth plate architecture and reserve, proliferative and hypertrophic zone dimensions did not differ between WT and GPB5KO mice at P42 (Supplemental Figure 2, A and B). Furthermore, linear growth velocity and final bone length were similar among male and female WT and GPB5KO mice (Supplemental Figure 2C). Decreased nasal and frontal bone length has previously been described in transgenic mice overexpressing GPB5 (21). However, no differences in skull morphology or dimensions were observed in thyrostimulin-deficient mice (Supplemental Figure 3).

#### Increased BMC, volume, and mineralization

**Figure 3.**
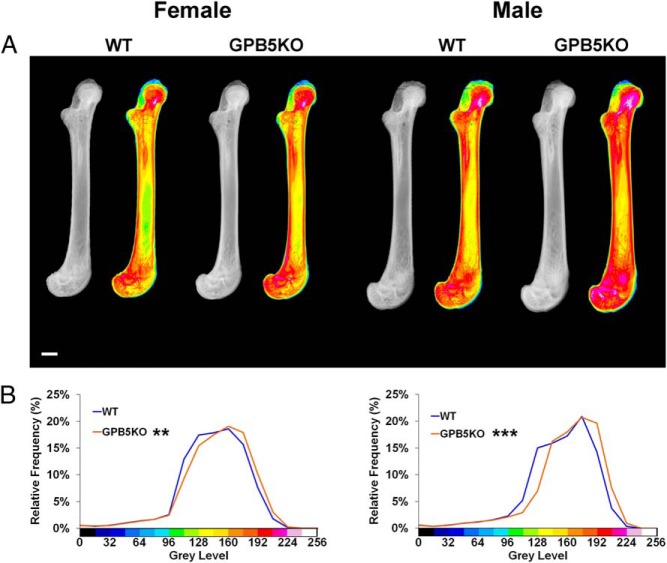
X-ray microradiography. A, Gray scale and pseudocolored images of femurs from P42 WT and GPB5KO mice. The gray scale pixel distribution was stretched between 0 and 256 levels relative to polyester, aluminum, and steel standards. Gray scale images were divided into 16 equal intervals, each represented by a different color to aid visual presentation of digital images. In these pseudocolored images, low mineral content is green and high mineral content pink. B, Relative frequency histograms of bone mineralization densities in which the gray scale pixel distribution is shown in relation to each of the 16 equally sized color bins (n = 4–6 per genotype, each gender). Kolmogorov-Smirnov test, GPB5KO vs WT; **, *P* < .01; ***, *P* < .001. Scale bar, 1000 μm.

Digital x-ray microradiography demonstrated increased BMC in juvenile GPB5KO mice compared with WT, and this difference was greater in males than females (Figure 3). Micro-CT and qBSE-SEM analyses were performed to determine whether the increased BMC was a consequence of increased bone volume, increased bone mineralization, or both.

**Figure 4.**
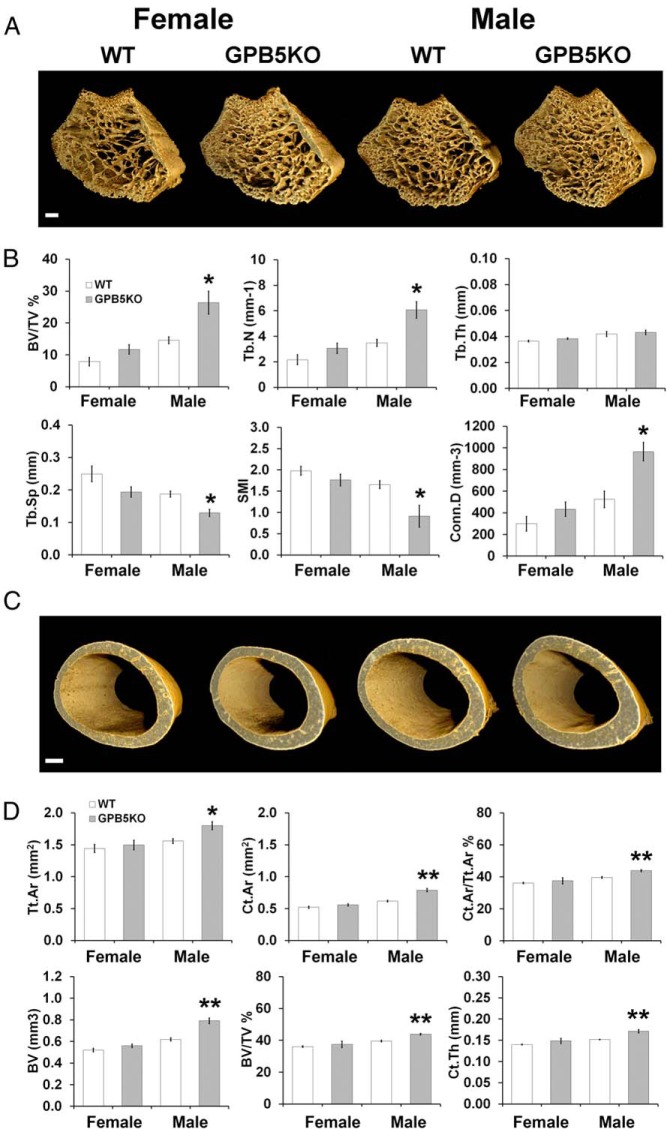
Micro-CT. A, Micro-CT images of the distal femoral metaphysis from P42 WT and GPB5KO mice. B, Graphs showing BV/TV, trabecular number (Tb.N), trabecular thickness (Tb.Th), trabecular spacing (Tb.Sp), structure model index (SMI) (an indicator of trabecular shape, in which pure rod-like shape = 3, pure plate-like = 0), and connectivity density (Conn.D) (mean ± SEM) in distal femur trabecular bone from P42 WT and GPB5KO mice. C, Micro-CT images of the middiaphyseal femur cortical bone from P42 WT and GPB5KO mice. D, Graphs showing total cross-sectional area of cortical bone inside the periosteal envelope (Tt.Ar), cortical bone area (Ct.Ar), cortical area fraction (Ct.Ar/Tt.Ar), cortical BV, cortical bone volume fraction (BV/TV), and average cortical thickness (Ct.Th) (mean ± SEM) in middiaphyseal femur from P42 WT and GPB5KO mice. Student's *t* test, GPB5KO vs WT; *, *P* < .05; **, *P* < .01 (n = 3 per genotype, per gender). Scale bars, 200 μm.

**Figure 5.**
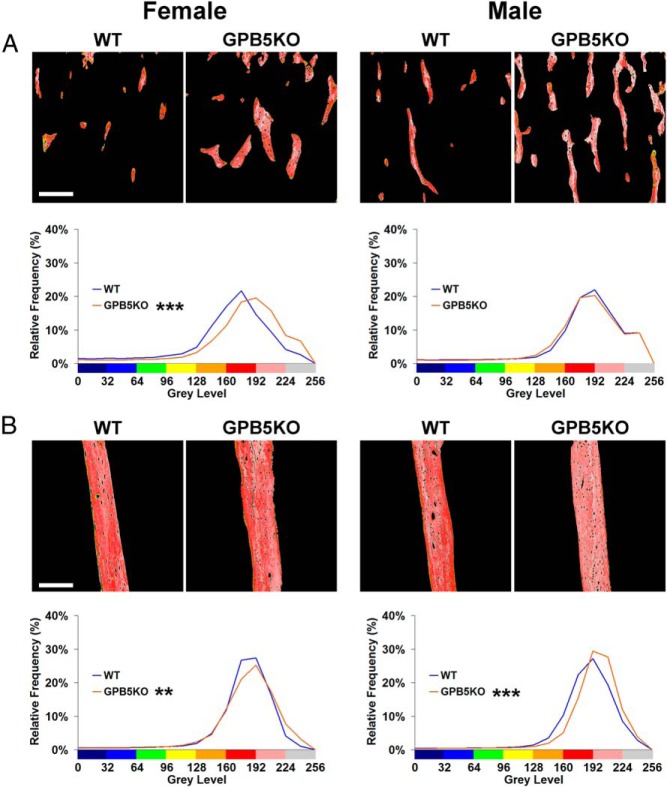
Bone micromineralization. A, qBSE-SEM gray scale and pseudocolored images of metaphyseal trabecular bone from P42 WT and GPB5KO mice. The gray scale pixel distribution was stretched between 0 and 256 levels relative to halogenated dimethacrylate standards as indicated in Materials and Methods. Gray scale images were divided into 8 equal intervals, each represented by a different color to aid visual presentation of digital images. In these pseudocolored images, low mineralization density is yellow and high density is gray. Graphs show relative frequency of micromineralization densities in which the gray scale pixel distribution is shown in relation to each of the 8 equally sized color bins. B, qBSE-SEM images and relative frequency histograms from middiaphyseal cortical bone in P42 mice. Kolmogorov-Smirnov test, GPB5KO vs WT; **, *P* < .01; ***, *P* < .001 (n = 3–5 per genotype, each gender). Scale bars, 200 μm.

Micro-CT analysis of femurs from juvenile animals demonstrated increased trabecular bone volume, number and connectivity, reduced trabecular spacing and more plate-like morphology in male GPB5KO mice (Figure 4, A and B). Trabecular bone parameters did not differ in female GPB5KO mice compared with WT. Juvenile male GPB5KO mice also had increased cortical bone area, volume and thickness but no differences were seen in females (Figure 4, C and D). Cortical bone porosity did not differ in juvenile GPB5KO mice of either gender compared with WT (male 1.15 ± 0.04% vs 1.07 ± 0.13%, *P* = .59; female 0.19 ± 0.04% vs 0.40 ± 0.13%, *P* = .19). qBSE-SEM demonstrated increased trabecular bone mineralization in juvenile female GPB5KO mice and increased cortical bone mineralization in juveniles of both genders (Figure 5).

#### Increased osteoblastic bone formation

**Figure 6.**
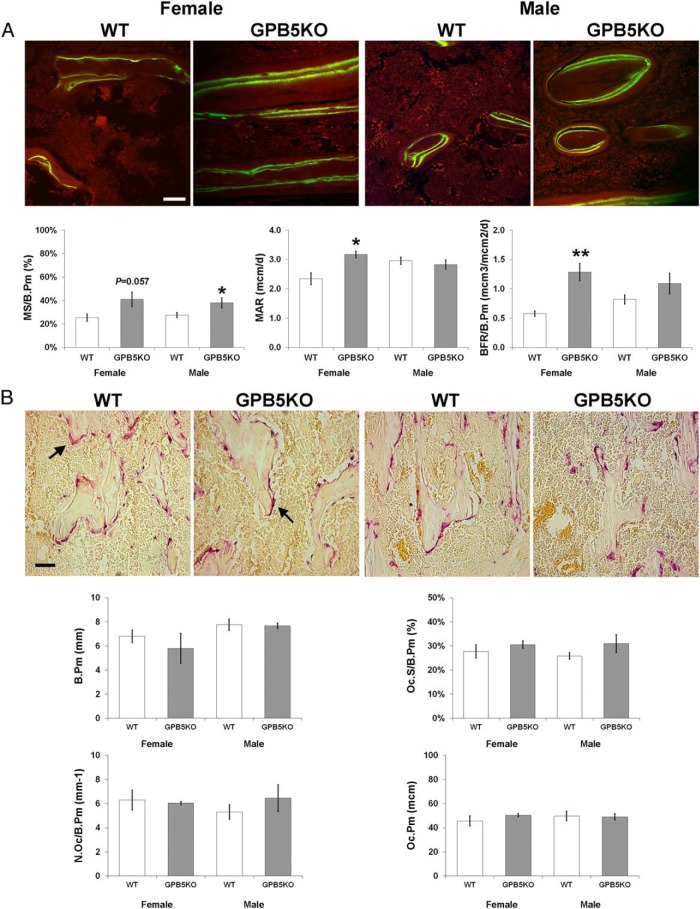
Dynamic and static histomorphometry. A, Confocal laser scanning microscopy (CLSM) images of calcein double labeled trabecular bone from P42 WT and GPB5KO mice. Scale bar, 50 μm. Two-dimensional quantitative analysis of mineralizing perimeter (MS/B.Pm), MAR, and BFR (BFR/B.Pm) (mean ± SEM). B, Proximal tibia trabecular bone sections from P42 WT and GPB5KO mice stained for TRAP activity. Arrows indicate TRAP positive osteoclasts. Scale bar, 100 μm. Two-dimensional quantitative analysis of the trabecular B.Pm, Oc.S/B.Pm, Oc.N/B.Pm, and the mean osteoclast perimeter (Oc.Pm). Student's *t* test, GPB5KO vs WT; *, *P* < .05; **, *P* < .01 (n = 3–5 per genotype, per gender, per age).

Static and dynamic histomorphometry was performed to investigate the cellular basis underlying the increased bone volume and mineralization in juvenile GPB5KO mice. Bone formation parameters were determined in samples from calcein double-labeled mice (Figure 6A). In juvenile female GPB5KO mice MAR (double calcein label separation divided by time between administration of labels) was increased by 26% and BFR (MAR multiplied by length of mineralizing surface) by 55%. In juvenile male GPB5KO mice, mineralizing surfaces were increased 27%, and BFR showed a similar trend to females. By contrast, osteoclastic bone resorption parameters did not differ among GPB5KO and WT mice (Figure 6B).

#### Normal bone strength and stiffness

Three-point bend testing was performed to characterize the functional consequences of thyrostimulin deficiency. Despite the increase in cortical bone thickness and mineralization in GPB5KO mice, biomechanical parameters (yield load, maximum load, fracture load, and stiffness) did not differ from WT animals (Supplemental Figure 4).

### Normalization of the skeletal phenotype in adult thyrostimulin-deficient mice

In contrast to the juvenile skeletal phenotype, adult GPB5KO mice had similar BMC to WT (Supplemental Figure 5). Minor increases in trabecular number and bone volume per tissue volume (BV/TV) were evident in females (*P* < .05 both parameters), and a small increase in cortical bone area (Ct.Ar/Tt.Ar) was present in males (*P* < .05). All other trabecular and cortical bone parameters did not differ between WT and GPB5KO mice of both genders (Supplemental Figure 6). Consistent with this, cortical bone porosity did not differ in adult GPB5KO mice compared with WT (male 0.70 ± 0.29% vs 1.10 ± 0.09%, *P* = .23; female 0.30 ± 0.33% vs 0.44 ± 0.22%, *P* = .61). BSE-SEM analysis of long bone microarchitecture was consistent with these x-ray microradiography and micro-CT findings (Supplemental Figure 7). In qBSE-SEM studies, only a small increase (*P* < .05) in cortical bone mineralization density was observed in adult female GPB5KO mice, whereas a small decrease (*P* < .05) was present in males (Supplemental Figure 8). In accord, bone formation and resorption parameters, and bone strength were similar among adult GPB5KO and WT mice (Supplemental Figures 4 and 9).

In summary, comprehensive skeletal phenotyping of GPB5KO mice using complementary approaches revealed that thyrostimulin deficiency results in a developmental phenotype characterized by a transient increase in bone formation and bone mass. Importantly, these abnormalities resolve by adulthood.

### TSH and thyrostimulin signaling in bone cells

To investigate the molecular mechanisms underlying the skeletal abnormalities identified in juvenile GPB5KO mice, the murine GPA2 and GPB5 subunits of thyrostimulin were expressed in COS-7 cells (Supplemental Figure 10A). Both GPA2 and GPB5 subunits were glycosylated and secreted into the medium, forming GPA2/GPB5 heterodimers and GPB5 homodimers (Supplemental Figure 10, B–D). Conditioned medium from COS-7 cells coexpressing GPA2 and GPB5, but not expressing GPA2 or GPB5 alone, induced a large cAMP response in TSHR-expressing CHO cells but no response in TSHR-deficient cells (Supplemental Figure 10E). Nevertheless, and despite strong cAMP responses to forskolin, neither hTSH nor thyrostimulin elicited a cAMP response in mouse primary osteoblasts, MC3T3 cells or mouse primary osteoclasts. Thus, in these studies cAMP responses to hTSH and thyrostimulin in osteoblasts and osteoclasts did not differ from controls (Supplemental Figure 10F).

**Figure 7.**
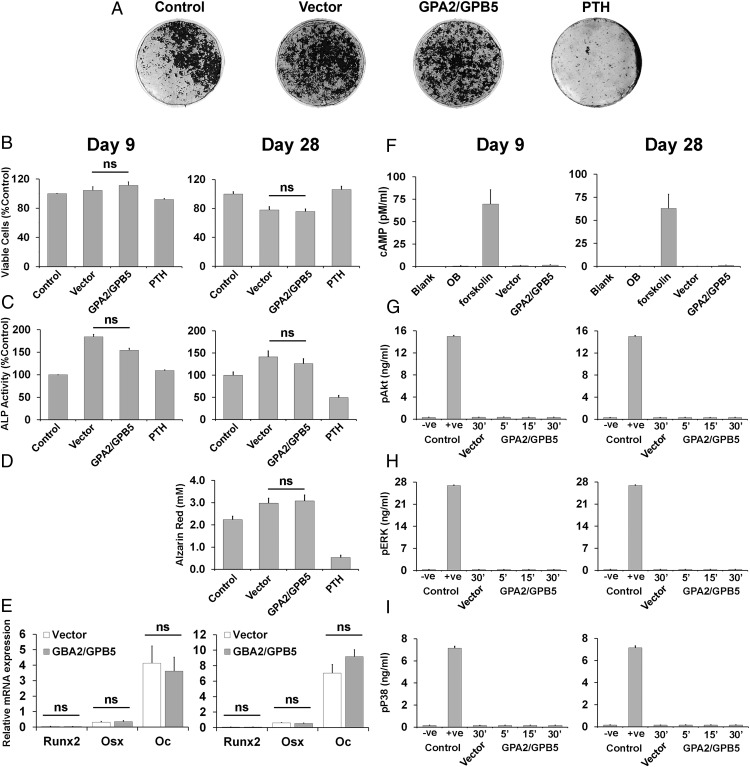
TSHR signaling in primary calvarial osteoblasts. A, Primary mouse osteoblasts treated for 28 days with medium (control), medium supplemented with conditioned medium from COS-7 cells (10% vol/vol) transfected with empty vector (vector), medium supplemented with conditioned medium from COS-7 cells (10% vol/vol) transfected with GPA2/GPB5 expression vector (GPA2/GPB5), and PTH (100μM). B, Graphs showing analysis of osteoblast proliferation (viable cells, %) after 9 and 28 days in response to treatments. C, Graphs showing analysis of osteoblast differentiation (ALP activity) after 9 and 28 days in response to treatments. D, Graphs showing analysis of osteoblast mineralization (alizarin red staining) after 9 and 28 days in response to treatments. (E) Graphs showing analysis of osteoblast gene expression (*Runx2*, *Osx*, and *Oc*) after 9 and 28 days in response to treatments. F, cAMP responses of primary osteoblasts cultured for 9 and 28 days after no treatment (OB) or in response to treatment with forskolin, conditioned medium from COS-7 cells (10% vol/vol) transfected with empty vector (vector), or conditioned medium from COS-7 cells (10% vol/vol) transfected with GPA2/GPB5 expression vector GPA2/GPB5. G–I, Analysis of noncanonical TSHR downstream signaling pathways in response to treatment with thyrostimulin (GPA2/GPB5) for 5, 15, and 30 minutes after 9 and 28 days: (G) Akt pathway activation determined by quantitation of phospho-Akt (pAkt); −ve control, MEM medium alone; +ve control, recombinant pAkt2; (H) ERK pathway activation determined by quantitation of phospho-ERK (pERK); −ve control, MEM medium; +ve control, recombinant pERK2; (I) P38 pathway activation determined by quantitation of phospho-P38 (pP38); −ve control, MEM medium; +ve control, recombinant pP38α.

To investigate whether thyrostimulin directly inhibits calvarial osteoblast function, cell proliferation, differentiation, and mineralization assays were performed (Figure 7). Primary calvarial osteoblasts were cultured for 9 and 28 days in the presence of conditioned medium from COS-7 cells transfected with vector alone or with medium from cells coexpressing GPA2 and GPB5. Continuous treatment with PTH was used as a positive control for inhibition of osteoblast activity. Thyrostimulin had no effect on the number of viable osteoblasts, ALP activity or nodule formation and mineralization at both time points (Figure 7, A–D) and did not affect levels of expression of *Runx2*, *Osx*, or *Oc* osteoblast marker gene mRNAs (Figure 7E). Persisting thyrostimulin activity was confirmed in the conditioned medium at completion of the 28-day culture period by determining the cAMP response in TSHR-expressing CHO cells (data not shown). To investigate whether thyrostimulin activates noncanonical TSHR downstream signaling pathways in calvarial osteoblasts, analysis of the Akt, ERK, and P38 (P38 MAPK) pathways was performed (Figure 7, F–I). Thyrostimulin had no effect on these downstream pathways in cells cultured for 9 or 28 days.

**Figure 8.**
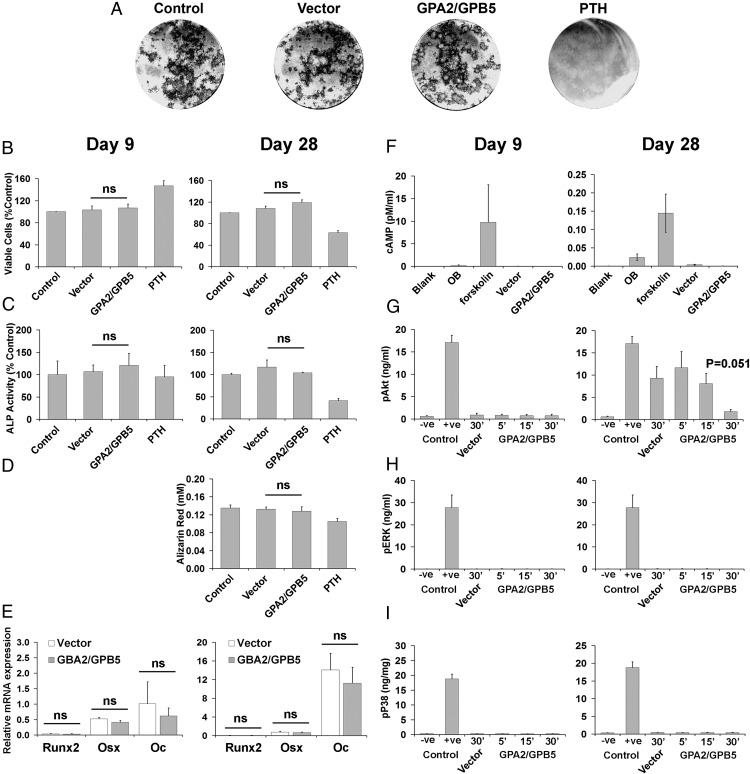
TSHR signaling in BMSC/osteoblast cultures. A, Primary mouse BMSC osteoblasts treated for 28 days with medium (control), medium supplemented with conditioned medium from COS-7 cells (10% vol/vol) transfected with empty vector (vector), medium supplemented with conditioned medium from COS-7 cells (10% vol/vol) transfected with GPA2/GPB5 expression vector (GPA2/GPB5), and PTH (100μM). B, Graphs showing analysis of cell proliferation (viable cells, %) after 9 and 28 days in response to treatments. C, Graphs showing analysis of osteoblast differentiation (ALP activity) after 9 and 28 days in response to treatments. D, Graphs showing analysis of osteoblast mineralization (alizarin red staining) after 9 and 28 days in response to treatments. E, Graphs showing analysis of osteoblast gene expression (*Runx2*, *Osx*, and *Oc*) after 9 and 28 days in response to treatments. F, cAMP responses of primary BMSC osteoblasts cultured for 9 and 28 days after no treatment (OB) or in response to treatment with forskolin, conditioned medium from COS-7 cells (10% vol/vol) transfected with empty vector (vector), or conditioned medium from COS-7 cells (10% vol/vol) transfected with GPA2/GPB5 expression vector GPA2/GPB5. G–I, Analysis of noncanonical TSHR downstream signaling pathways in response to treatment with thyrostimulin (GPA2/GPB5) for 5, 15, and 30 minutes after 9 and 28 days: (G) Akt pathway activation determined by quantitation of phospho-Akt (pAkt); −ve control, MEM medium alone; +ve control, recombinant pAkt2; (H) ERK pathway activation determined by quantitation of phospho-ERK (pERK); −ve control, MEM medium; +ve control, recombinant pERK2; (I) P38 pathway activation determined by quantitation of phospho-P38 (pP38); −ve control, MEM medium; +ve control, recombinant pP38α.

To investigate further whether the effects of thyrostimulin on bone formation are likely to be mediated by primary actions on osteoblast precursor cells, studies were also performed in BMSC/osteoblasts cultured for 9 and 28 days (Figure 8). Thyrostimulin had no effect on the number of viable BMSC osteoblasts, ALP activity, nodule formation, mineralization or marker gene expression at both time points (Figure 8, A–E). Thyrostimulin also had no effect on noncanonical TSHR downstream signaling pathways (Figure 8, F–I), although there was a trend for inhibition of AKT activation after 30 minutes treatment (*P* = .051) in day-28 BMSC osteoblasts (Figure 8G, right panel).

## Discussion

During postnatal skeletal development the ancestral glycoprotein hormone subunits *Gpa2* and *Gpb5* were transiently expressed in long bones (formed by endochondral ossification) and the skull (formed by intramembranous ossification). However, the levels of *Gpa2* and *Gpb5* mRNAs were variable in cultured osteoblasts and osteoclasts, whilst currently available antibodies were not sufficiently sensitive or specific to determine expression of GPA2 and GPB5 proteins (data not shown).

Consistent with the transient skeletal expression of thyrostimulin during early postnatal development, the abnormal phenotype in juvenile GPB5KO mice resolved by early adulthood. Skeletal findings in GPB5KO mice were restricted to abnormalities of bone formation and mineralization, demonstrating a primary role for thyrostimulin in the regulation of osteoblast activity. Importantly, linear growth and long bone modeling and morphology were unaffected in GPB5KO mice, indicating that thyrostimulin deficiency does not affect chondrocyte or osteoclast function.

Comprehensive analysis of the skeleton using complementary techniques revealed similar phenotypes in males and females, with small temporal differences observed between genders. In juvenile GPB5KO mice, trabecular and cortical bone mass were increased in males but not females. Females, however, displayed a higher BFR and increased trabecular bone mineralization at a young age but had increased trabecular bone volume persisting to adulthood. Previous studies did not determine thyroid status separately in males and females but showed that juvenile GPB5KO mice have transient hypothyroxinemia with a compensatory increase in TSH (17). In the current studies, juvenile female GBP5KO mice and adults of both genders were euthyroid, whereas juvenile males had a trend for hypothyroxinaemia (*P* = .06) with an increased TSH. The similar TSH response to TRH in juvenile GPB5KO and WT mice revealed intact pituitary thyrotroph responsiveness in GPB5KO mice, suggesting that the transient hypothyroxinemia in males is a consequence of altered thyroid hormone synthesis or metabolism.

Overall, the skeletal consequences of thyrostimulin deficiency appear to occur earlier and resolve more rapidly in males than females, and it is important to consider whether this may result in part from gender differences in systemic T_4_ concentrations. Importantly, impaired chondrocyte differentiation and linear growth are the most sensitive markers of reduced thyroid hormone action in the developing skeleton (34, 37, 42, 43) and juvenile hypothyroidism also results in decreased osteoblastic bone formation, reduced bone volume and impaired bone mineralization (34, 37, 42). Because growth parameters were unaffected in GPB5KO mice, and osteoblast function was increased rather than decreased, the reduction in T_4_ observed in males during growth is unlikely to contribute to the skeletal abnormalities observed. Furthermore, similar skeletal abnormalities were observed in euthyroid juvenile females, emphasizing the phenotype is independent of altered systemic thyroid status. By contrast, the increased osteoblastic bone formation, bone volume, and mineralization observed in juvenile GBP5KO mice correlates closely with the transient expression of thyrostimulin in the developing skeleton. Together, these considerations identify thyrostimulin as a negative regulator of osteoblastic bone formation during skeletal development.

To determine the mechanism of thyrostimulin action we coexpressed recombinant GPA2 and GPB5 subunits and generated functionally active thyrostimulin, which potently activated a canonical cAMP response in TSHR-expressing CHO cells. However, thyrostimulin, GPA2, GPB5, or TSH all failed to activate cAMP in osteoblasts and osteoclasts despite expression of the TSHR. The lack of response to TSH is consistent with previous studies (23, 24, 44), but we now demonstrate that even thyrostimulin, a 4-fold more potent ligand for TSHR, also fails to activate cAMP in osteoblasts and osteoclasts. We next investigated whether thyrostimulin acts via noncanonical TSHR signaling pathways in calvarial osteoblasts and BMSC-derived osteoblast precursors. In both cell types, thyrostimulin failed to activate Akt, ERK or P38 and did not affect osteoblast proliferation, differentiation, mineralization or marker gene expression. Overall, these data demonstrate the effects of thyrostimulin on bone formation are indirect and not cell autonomous in osteoblast precursor cells or mature osteoblasts.

In conclusion, these studies demonstrate a transient role for thyrostimulin during early skeletal development. The predicted limited stability of GPA2/GPB5 heterodimers when compared with other glycoprotein hormones (18) suggests these actions are local rather than systemic, although the cellular origin and target cells of thyrostimulin in bone remain uncertain. Nevertheless, similar transient developmental effects of thyrostimulin have been suggested in other tissues (1, 4, 17, 19, 20) and species (5, 15–17), indicating that this ancient glycoprotein may have diverse developmental roles in both vertebrates and nonvertebrates. Importantly, both thyrostimulin and the TSHR are expressed in bone and the current studies indicate that thyrostimulin has retained a developmental function but its actions are dispensable in the adult skeleton. Consistent with such a role, activation of TSHR signaling has been implicated in self-renewal, differentiation, and survival of various progenitor and stem cell populations (45–49). Overall these considerations suggest a possible role for thyrostimulin in the regulation of tissue development and repair. Future studies will be required to investigate this novel field.
